# Human striatum is differentially activated by delayed, omitted, and immediate registering feedback

**DOI:** 10.3389/fnhum.2012.00243

**Published:** 2012-08-30

**Authors:** Christin Kohrs, Nicole Angenstein, Henning Scheich, André Brechmann

**Affiliations:** ^1^Special Lab Non-Invasive Brain Imaging, Leibniz Institute for NeurobiologyMagdeburg, Germany; ^2^Department of Auditory Learning and Speech, Leibniz Institute for NeurobiologyMagdeburg, Germany

**Keywords:** feedback, medial prefrontal cortex, timing, striatum, prediction error

## Abstract

The temporal contingency of feedback during conversations is an essential requirement of a successful dialog. In the current study, we investigated the effects of delayed and omitted registering feedback on fMRI activation and compared both unexpected conditions to immediate feedback. In the majority of trials of an auditory task, participants received an immediate visual feedback which merely indicated that a button press was registered but not whether the response was correct or not. In a minority of trials, and thus unexpectedly, the feedback was omitted, or delayed by 500 ms. The results reveal a response hierarchy of activation strength in the dorsal striatum and the substantia nigra: the response to the delayed feedback was larger compared to immediate feedback and immediate feedback showed a larger activation compared to the omission of feedback. This suggests that brain regions typically involved in reward processing are also activated by non-rewarding, registering feedback. Furthermore, the comparison with immediate feedback revealed that both omitted and delayed feedback significantly modulated activity in a network of brain regions that reflects attentional demand and adjustments in cognitive and action control, i.e., the posterior medial frontal cortex (pMFC), right dorsolateral prefrontal cortex (dlPFC), bilateral anterior insula (aI), inferior frontal gyrus (Gfi), and inferior parietal lobe (Lpi). This finding emphasizes the importance of immediate feedback in human–computer interaction, as the effects of delayed feedback on brain activity in the described network seem to be similar to that of omitted feedback.

## Introduction

A mutual exchange of information can be described as a dialog, performed by at least two participants. During communication, the conversational partners need to achieve a common ground to ensure that a listener understood the speaker's message and intention. Language as well as gestures and body language are used to satisfy this expectation during human conversation (Clark and Brenan, 1991). However, dialogs are not limited to human–human interactions but, from the point of view of a user, their structure is also realized in human–computer interactions. In all these situations, feedback serves to fulfill the need for closure, the subjective sense of completion (Miller, 1968). Even today—after decades of stunning enhancements in computer science—research on system response time has not lost its topicality. The return to network-based computing caused new problems in delivering data in time due to the fact that nowadays internet-based applications depend on the level of performance by the network (Dabrowski and Munson, 2011).

From human–computer interaction it is known that dialogs should be conducted in real time, otherwise participants may get irritated (Shneiderman and Plaisant, 2005). If the user obtains no response after initiating an action he will face a basic question:—was my action registered or—must I repeat it? (Pérez-Quinones and Sibert, 1996). The response time of a computer during a human–computer interaction can vary, depending on the complexity of the action requested by the user. However, there is large inter-individual variation in acceptable waiting time, depending on many factors, such as personality, age, mood, cultural context, time of day, or noise (Shneiderman and Plaisant, 2005). For simple repetitive tasks that require little problem solving, users want to perform the task rapidly and are irritated by delays of more than a few tenths of a second (Shneiderman and Plaisant, 2005). During such a simple task, i.e., an illumination after pressing a button to call an elevator, users expect a response within 200 ms (Miller, 1968). While humans are highly adaptive and are able to habituate to a fixed delay, an unexpected delay of a response will always be disruptive (Shneiderman and Plaisant, 2005).

In the current fMRI experiment, we studied the effect of such an unexpected delay in feedback presentation on brain activation. To this avail, we used neutral registering feedback, i.e., feedback that only informs the subjects that a button press was registered without evaluating whether their decision in the auditory categorization task was right or wrong.

Several imaging studies describe a network of brain areas involved in feedback processing. These areas could be candidate regions whose activity may be modulated when a feedback is presented with an unexpected delay. However, most studies analyzed the influence of positive and negative feedback (reward and punishment) (see O'Doherty, 2004; Delgado et al., 2005; Knutson and Cooper, 2005; Nieuwenhuis et al., 2005; Marco-Pallares et al., 2007). Current research on feedback processing focuses either on dopaminergic brain regions or areas that receive strong dopaminergic and thus reward-related input (Redgrave and Gurney, 2006; Düzel et al., 2009). The response of such regions to basic registering feedback, however, remains unclear. For example, Nieuwenhuis et al. (2005) presented a “+” for correct and a “−” for incorrect responses, and in addition a “?” as uninformative and therefore non-rewarding feedback. But even though registering, non-rewarding feedback was presented in this study, it was used as control condition and only in conjunction analysis with positive and negative feedback. Only one previous study by Behne et al. (2008) found direct evidence that the dorsal striatum is already activated by non-rewarding feedback. The authors argue that temporally contingent feedback which merely indicates the registration of a subject's button-press “constitutes the basic framework by which the brain recognizes that this is a dialog situation” (Behne et al., 2008, p. 1497).

Besides areas that are directly involved in feedback processing, those brain regions that respond to prediction errors are likely to be modulated by unexpectedly delayed feedback. In a study using electroencephalography (EEG), Holroyd et al. (2006) showed that neutral feedback elicits a feedback error-related negativity (fERN) as large as that of negative feedback, when participants expect a positive feedback. Based on this result they proposed that the evaluative system underlying the fERN classifies outcomes into those that indicate that a goal was fulfilled and those that do not (Holroyd et al., 2006). The fERN is localized in medial frontal areas [dorsal anterior cingulate cortex, medial prefrontal cortex, and posterior medial frontal cortex (pMFC)] but compared to the well characterized ERN, this component seems to be more widely distributed over the scalp (Müller et al., 2005; Nieuwenhuis et al., 2005). The medial prefrontal cortex was recently found to be activated not only after violations of predicted outcome valence but also by violations of predicted outcome timing (Forster and Brown, 2011). According to Holroyd et al. (2006), the involvement of the regions in medial frontal areas that contribute to the fERN also depends on midbrain dopamine signals. While a decreasing dopamine signal after the omission of a feedback (negative reward prediction error) would lead to a stronger fERN, an increase of dopamine activity after a positive feedback (positive reward prediction error) would lead to a decreased fERN (Ridderinkhof et al., 2004). Hollerman and Schultz (1998) proposed that midbrain dopamine neurons code not only errors in the prediction of occurrence but also errors in the timing of rewarding feedback. Dopamine neurons showed a positive response when a reward unexpectedly occurred at an unpredicted time and a negative response when a reward failed to occur at the predicted time. However, Redgrave and Gurney (2006) suggest that, rather than predicting the occurrence of reward, phasic dopamine release has a role in the reselection of actions after an unpredicted event.

In the current study we used an auditory categorization task in which participants received immediate registering feedback in the majority (76%) of trials. Therefore, we supposed that the participants expected the registering feedback after each button press and that they expected it to occur immediately. The first aim of our study was to characterize the neuronal effects of unexpectedly delayed feedback (500 ms delay, 12% of the trials) compared to unexpectedly omitted feedback (12% of the trials). Based on the work of Holroyd et al. (2006) on fERN, we assumed that the unexpected omission of registering feedback leads to strongest neuronal activity in medial frontal areas suggested to participate in the generation of the fERN. Due to the fact that in the delayed condition a feedback did occur, albeit with a moderate delay of 500 ms, we expected less activity in this condition compared to the omission of feedback. Based on the hypothesis that the fERN also depends on midbrain dopamine signals (Holroyd et al., 2006) and our previous finding that the dorsal striatum may already be activated by immediate registering feedback (Behne et al., 2008), we asked the question whether the dopaminergic system is involved in the processing of omitted and delayed registering feedback. If registering feedback indeed leads to activation of the dopaminergic system, we should find a stronger activity in the striatum and SN/VTA after unexpected delays of feedback as compared to unexpected omissions of feedback which has been shown to suppress dopaminergic activity (Hollerman and Schultz, 1998; McClure et al., 2003; D'Ardenne et al., 2008).

## Materials and methods

### Participants

In the present study, 17 right-handed participants [Edinburgh Handedness Inventory, (Oldfield, 1971)] with normal hearing participated. The averaged laterality quotient was 87.3 ± 13.4 with a range from 69 to 100. Participants (9 females and 8 males, aged 21–44 years, mean age 27 years) gave written informed consent to the study, which was approved by the ethics committee of the University of Magdeburg. Two participants were removed from further analysis because of motion artifacts (more than 3° or 2.5 mm) and one because they reported that they did not attend the feedback throughout the entire experiment. One participant exceeded the error criterion (less than 60% correct for both classification conditions) and was also excluded. Thus, the data of 13 subjects were used for the group level analysis.

### Stimuli and task

Linearly frequency modulated (FM) tones with a duration of 600 ms served as acoustic stimuli. The FM tones differed in direction of frequency modulation (20 upward, 20 downward) and in center frequency (*F*_*C*_ = 1100−3000 Hz in steps of 100 Hz) with starting and end frequency calculated by

$$F_{C}(\text{Hz})\pm F_{C}(\text{Hz})/2\times \Delta t\;(\text{s}).$$

The 198 FM tones were presented pseudo-randomly in an event-related design with a jittered intertrial interval of 6, 8, and 10 s.

The participants were told that they had to categorize the FM tones according to the direction of modulation (Behne et al., 2005; Brechmann and Scheich, 2005). They had to press a button with the right index finger in response to upward modulated FM tones and another button with their right middle finger indicating downward modulated FM tones. Participants were told that the feedback only registers their button press independent of the correctness of their response.

During the entire experiment participants had to look at a white fixation cross on a black computer screen. Stimuli were back-projected onto the screen which could be viewed via a mirror mounted on the head coil. The distance between the participants' eyes and the screen was 59 cm. The screen was 325 × 260 mm, which is appropriate for a visual angle of about ±15°. The font size used for presenting the feedback was 62 (Arial). After pressing the button, they received an uninformative registering visual feedback, which only indicated the registration of the button press and did not inform the participants about the correctness of their response. This visual feedback was presented for 500 ms. If the participants answered within 1.5 s after FM tone onset they received a green checkmark, indicating that they answered fast enough. If they were too slow they saw a red cross. In the vast majority of trials (98.5%) the 1.5 s interval was long enough to respond. Therefore, we decided to exclude the few trials that were too slow from further analysis.

The 198 FM stimuli were presented in three randomly distributed trial conditions in one session. In the first condition, participants received the feedback immediately after the button press. In a second condition, the feedback was presented with a delay of 500 ms. In the third condition, no feedback was presented. In most trials participants received the feedback immediately (76%). The other two conditions occurred only infrequently (12% each). Participants were not informed about the delay condition but they were told that any omission of feedback is only an infrequent problem that is not part of the experiment.

After the fMRI experiment, subjects were asked to fill in a short questionnaire. First they were asked about their subjective difficulty of performing the categorization task on a scale ranging from 1 (very easy) to 7 (very difficult). They also had to assess if they attended to the feedback throughout the entire experiment. Furthermore, they had to report if the feedback appeared immediately after their button presses at all times. We asked them if the omission of feedback elicited any emotional or behavioral response. Finally, we asked if they noticed a delay in feedback presentation and if this delay elicited any emotional or behavioral response.

### Data acquisition

The measurements were carried out in a 3 Tesla scanner (Siemens Trio, Erlangen, Germany) equipped with an eight channel head coil. A 3D anatomical data set of the participant's brain (echo time (TE), 4.77 ms; repetition time (TR), 2500 ms; flip angle, 7°; matrix size, 256 × 256; field of view, 25.6 × 25.6 cm; 192 slices of 1 mm each) was obtained before the functional measurement. Additionally, an *Inversion-Recovery-Echo-Planar-Imaging* (IR-EPI) was acquired that has the same geometric distortions as the functional measurement but a reversed contrast and thus serves the purpose of a more precise coregistration of the functional data to the anatomical data. For fMRI, 828 functional volumes were acquired in 27 min and 36 s using an echo planar imaging (EPI) sequence (TE, 30 ms; TR, 2000 ms; flip angle, 80°; matrix size, 64 × 64; field of view, 19.2 × 19.2 cm; 32 slices of 3 mm thickness with 0.3 mm gaps).

The head of the participant was fixed with a cushion with attached ear muffs containing the fMRI compatible headphones (Baumgart et al., 1998). Additionally, the participants wore earplugs. The software Presentation (Neurobehavioral Systems, Albany, USA) was used for stimulus presentation and recording behavioral responses. Before the experiment, the overall stimulus intensity was adjusted for each participant to a comfortable level and equally loud at both ears. Visual stimuli were presented by a video projector onto a back projection screen, which was visible inside the scanner via a mirror system.

### Data analysis

The functional data were analyzed with the software BrainVoyager™QX (Brain Innovation, Maastricht, The Netherlands). A standard sequence of preprocessing steps, i.e., slice scan time correction, 3D-motion correction, linear trend removal, spatial smoothing with a Gaussian filter with 4 mm full width at half maximum, and filtering with a high-pass of three cycles per scan was performed. The functional data were co-registered with the 3D anatomical data by using the IR-EPI, and then transformed into Talairach-space (Talairach and Tournoux, 1988).

A deconvolution analysis was performed (Dale and Buckner, 1997), because the stimulus presentation rate was faster than the return to base level of the blood oxygenation dependent-signal (BOLD-signal), which may cause an overlap of the BOLD response between the three conditions at a certain time point (*t*). The gray value (*y*) at a time point (*t*) is the sum of the three overlapping conditions and a baseline condition [mean gray value (k)].

The deconvolution analysis is based on a linear model:

y=Xβ+ε

where *y* describes the measured grayscale value for the time points (*t* = 1−828), *X* is the design matrix with entries of 0 or 1 that describes the relationship between stimulus presentation and the observed data. The estimated beta values (β) describe the relationship between *y* and *X*, and ε is the error term.

The functional data were z-transformed. In the deconvolution analysis, the hemodynamic response is not estimated from a fixed function (like a γ-function) but is flexibly and adaptively estimated from the data. The deconvolution analysis models the hemodynamic response function based on the points in time *t* when a stimulus is presented, under the assumption of linearity and a finite number of data points of the response as predictors. For each condition ten points in time (18 s, predictors) were defined. The resulting design matrix *X* consisted of 31 columns, ten columns (predictors) for each condition and the constant factor k (mean gray value) that represented the baseline. Accordingly, 31 beta weights were estimated that allowed reconstructing the hemodynamic BOLD response for each condition. Because of the fast stimulus presentation rate, a correction for serial correlation was performed.

To identify the regions with differential BOLD responses, we compared the conditions at the conjoined time points 4 and 5 in a random effects analysis. Thus, the analysis focused on the period from 6 to 10 s after stimulus onset which represented the maximum BOLD response to both the stimulus and the immediate and delayed feedback. The reported general linear model (GLM) parameters (beta weights) provide a direct estimate of the actual percent signal change.

To identify regions that were differentially activated by delayed vs. omitted feedback, we computed a contrast wherein the activation had to be significantly higher to delayed feedback than to omitted feedback (FDR: *t* = 5.54, *q* = 0.05). In a further step, we analyzed if dopaminergic brain regions like the substantia nigra are activated during delayed and immediate feedback. Therefore, we compared delayed and immediate feedback versus baseline (FDR: *t* = 4.14, *q* < 0.05). The baseline is defined as the average BOLD amplitude across the whole scan which is dominated by intertrial time.

To identify regions that were activated in common by the two unexpected feedback conditions, we determined voxels that were activated in the balanced contrast of the two unexpected feedback conditions (delayed and omitted) versus the immediate feedback condition (FDR: *t* = 4.39, *q* = 0.05).

Volumes-of-interest (VOIs) were all resulting clusters that comprised at least 20 mm^3^. The size of these VOIs was determined by counting the number of enclosed voxels. For each VOI, a random effects ROI-GLM (region of interest-general linear model) as is implemented in BrainVoyager was conducted to determine the mean beta-value of each condition.

As two of the participants reported that they had not noticed any delayed feedback, we in addition analyzed them separately and computed a fixed effects analysis with the same contrasts as in the group analysis using a *t*-value >3 to generate hypotheses about potential deviations from the group activity pattern which may reflect that these subjects did not recognize any delayed feedback.

## Results

### Behavioral data

The average reaction time of the 13 participants was 815 ms (SD = ±171.5 ms) after FM tone onset and therefore significantly below the maximum response time of 1500 ms after FM tone onset. Only in a few trials (1.2% SD = ±1.4%) participants did not answer in time. These trials were excluded from further calculations. The average error rate in the directional categorization task was very low with 9.98% (SD = ±10.86%). Furthermore the average subjective difficulty of solving the categorization task (measured in a questionnaire after the fMRI experiment) is only 2.8 (SD = ±1.3) (on a scale from 1, very easy, to 7, very difficult).

Subjects who stated in the questionnaire that they did not attend to the feedback during the entire experiment were excluded from further analysis. No significant differences in reaction time or error rate were found between trials following delayed and omitted feedback compared to trials following immediate feedback. At the beginning of the experiment, participants tended to press the button more than once when feedback was omitted. Four participants pressed the button twice in one trial, two participants in two trials, three participants in three trials and two participants in four trials.

Six participants reported in the questionnaire that they felt irritated by the omission of feedback, the rest of them did not report any emotional changes when feedback was not presented immediately.

### Imaging data

**Figure 1:**
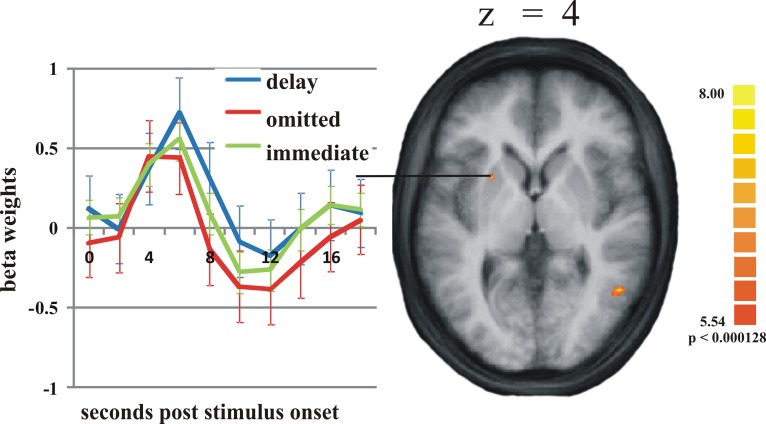
**Activation differences in the putamen.** On the right: the activation cluster of the putamen in the whole brain analysis comparing delayed and omitted feedback (*q* = 0.05). On the left: the time course of the BOLD response in this cluster in response to delayed feedback (blue line), immediate feedback (green line), and omitted feedback (red line). Error bars indicate standard error of the mean (SEM).

On the selected level of significance (corrected for multiple comparisons (FDR): *q* = 0.05), the comparison between delayed and omitted feedback revealed only two regions that were more strongly activated after delayed than after omitted feedback (Table 1). In the visual cortex (BA 19) we found significantly less activity for omitted feedback, most likely due to the missing visual input. Furthermore, and in accordance with our first hypothesis, the direct contrast also revealed a significant effect in the left putamen. When separately comparing this region's BOLD response time courses elicited by delayed feedback, omitted feedback and immediate feedback, we found that the response to delayed feedback was significantly stronger compared to the omission of feedback as well as compared to immediate feedback. The BOLD response for omitted feedback, however, was smaller than for immediate feedback (see Table 2 and Figure 1).

**Table 1:** **Brain areas with stronger BOLD signal after delayed compared to omitted feedback (*q = 0.05*)**.

**Region of activation**	**Talairach coordinates**						
	**Hemisphere**	**BA**	***x***	***y***	***z***	**Volume (mm^3^)**	**Mean *t***
**Putamen**	Left		−24	9	3	20	6.04
**Inferior temporal gyrus**	Right	19	47	−55	−0	903	6.54

**Table 2:** **ROI analysis of brain areas with stronger BOLD signal after delayed compared to omitted feedback**.

**Region**	**Contrast**	***t***	***p***
**Putamen**	Delay vs. Omitted	**7.63**	**0.00**
(Left hemisphere)	Delay vs. Immediate	**3.70**	**0.00**
	Immediate vs. Omitted	**3.15**	**0.01**
**iTG**	Delay vs. Omitted	**9.27**	**0.00**
(Right hemisphere)	Delay vs. Immediate	2.65	0.02
	Immediate vs. Omitted	**8.28**	**0.00**

Significant values are marked in bold.

**Figure 2:**
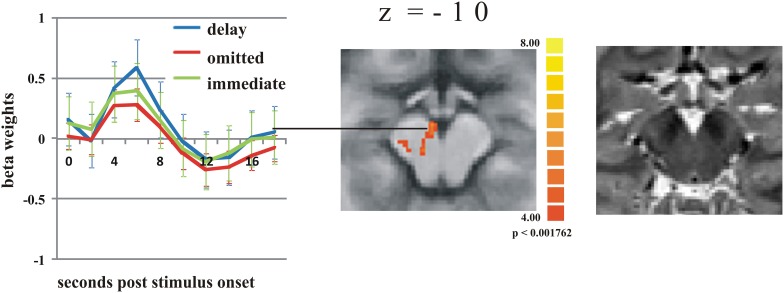
**Activation differences in the substantia nigra.** The strongest activation can be found during delayed feedback (blue line) compared to immediate feedback (green line), and omitted feedback (red line) (*q* < 0.05) (see Table 3 for statistical ROI analysis). Error bars indicate SEM. The T2 weighted image of the SN/VTA regions on the right site clearly shows the dark regions of SN.

To test whether the response in the putamen is potentially due to dopaminergic modulation, we analyzed the time course of activation in a midbrain region with the Talairach coordinates of −4(*x*) −15(*y*) −10(*z*) that corresponds to the substantia nigra/ventral tegmental area (SN/VTA) according to the “Talairach daemon” (Lancaster et al., 2000). This is supported by Figure 2 showing a T2-weighted image of a subject's brainstem area, transformed into Talairach space. Figure 2 also shows the activation cluster (70 mm^3^) that was significant (*q* < 0.05) comparing delayed and immediate feedback versus baseline (*mean* t = 4.66). We then calculated a ROI-GLM comparing all three conditions in the SN/VTA which revealed a similar response hierarchy of the BOLD activity as in the putamen, with significantly stronger activity during delayed compared to omitted feedback (see Table 3).

**Table 3:** **ROI analysis of the SN/VTA**.

**Region**	**Contrast**	***t***	***p***
**SN/VTA**	Delay vs. Omitted	**3.13**	**0.01**
(Left hemisphere)	Delay vs. Immediate	**3.41**	**0.01**
	Immediate vs. Omitted	1.36	0.20

Significant values are marked in bold.

**Figure 3:**
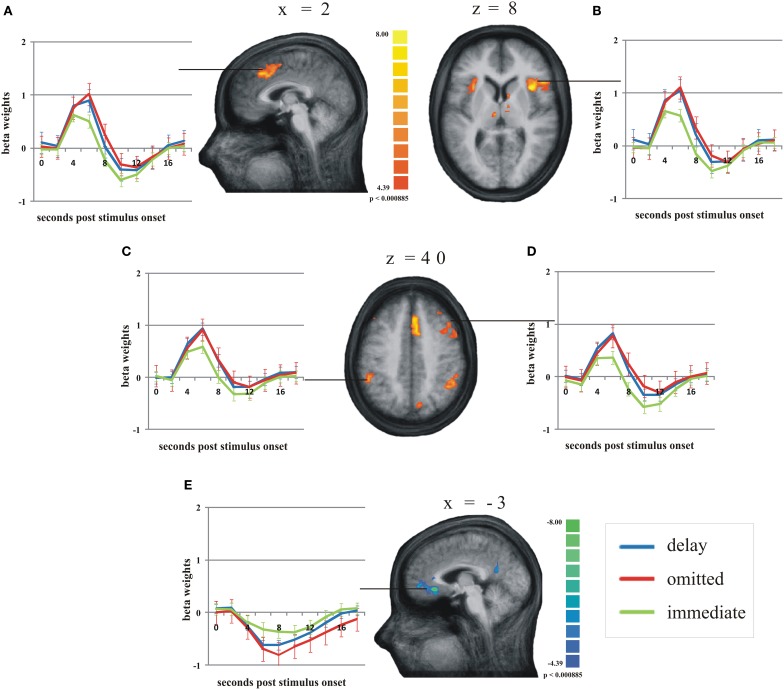
**Main regions of the group analysis with significant effects (activation/deactivation) during delayed (blue line) and omitted feedback (red line) compared to immediate feedback (green line) (*q* = 0.05).** Error bars indicate SEM. **(A)** posterior medial frontal cortex; **(B)** right anterior insula/inferior frontal gyrus; **(C)** left inferior parietal lobe; **(D)** right dorsolateral prefrontal cortex; **(E)** anterior cingulate gyrus.

According to our second hypothesis, we expected a number of brain areas to be more strongly activated by omitted feedback than by delayed feedback, especially those brain regions known to respond to prediction errors like the pMFC. As this was not the case, we performed a balanced contrast between delayed and omitted feedback on the one hand and immediate feedback on the other hand to test whether both unexpected feedback conditions led to comparably strong activation differences in those brain regions known to respond to prediction errors. This revealed a number of brain regions stronger activity during both unexpected feedback conditions compared to immediate feedback (see Table 4 for a complete list of regions). The largest differences in terms of the number of activated voxels were found in the pMFC, right dorsolateral prefrontal cortex (dlPFC), bilateral anterior insula/ inferior frontal gyrus (aI/Gfi) and inferior parietal lobe (Lpi) (Figure 3). Note that in this contrast there was an overall bias toward the right hemisphere. In the anterior and posterior cingulate cortex (ACC/PCC) (Figure 3) the BOLD signal during any condition decreased compared to baseline. Delayed and omitted feedback led to a significantly stronger decrease than immediate feedback.

**Table 4:** **Brain areas with stronger BOLD signal during delayed and omitted feedback compared to the immediate feedback condition (*q* = 0.05)**.

	**Talairach coordinates**						
	**Hemisphere**	**BA**	***x***	***y***	***z***	**Volume (mm^3^)**	**Mean *t***
**REGION OF ACTIVATION**							
**Medial frontal gyrus**	Left/Right	6/8	5	18	43	2590	5.40
**Anterior insula**	Right	13	41	18	5	4015	5.67
**Anterior insula**	Left	13	−30	18	5	1153	5.13
**Dorsolateral prefrontal gyrus**	Right	9/10	38	42	27	888	4.94
**Precentral gyurs**	Right	9	43	26	34	1262	5.10
**Middle frontal gyrus**	Right	9	45	12	36	2422	5.18
**Middle frontal gyrus**	Left	9	−41	29	37	67	4.72
**Middle temporal gyrus**	Right	21	56	−25	−5	191	4.86
**Inferior parietal lobe**	Right	40	48	−44	37	2744	5.42
**Inferior parietal lobe**	Left	40	−46	−37	41	595	5.05
**Precuneus**	Right	7	10	−68	39	299	5.01
**Nucleus caudatus**	Right		13	8	12	224	5.38
**Thalamus**	Right		10	−12	10	113	4.82
**Thalamus**	Left		−7	−16	9	131	5.01
**REGION OF DEACTIVATION**							
**Anterior cingulate gyrus**	Left/Right	32	−2	36	−3	3226	−5.23
**Superior frontal gyrus**	Left	8	−17	26	45	315	−4.90
**Posterior cingulate gyrus**	Left/Right	30	−2	−53	20	560	−5.21

**Figure 4:**
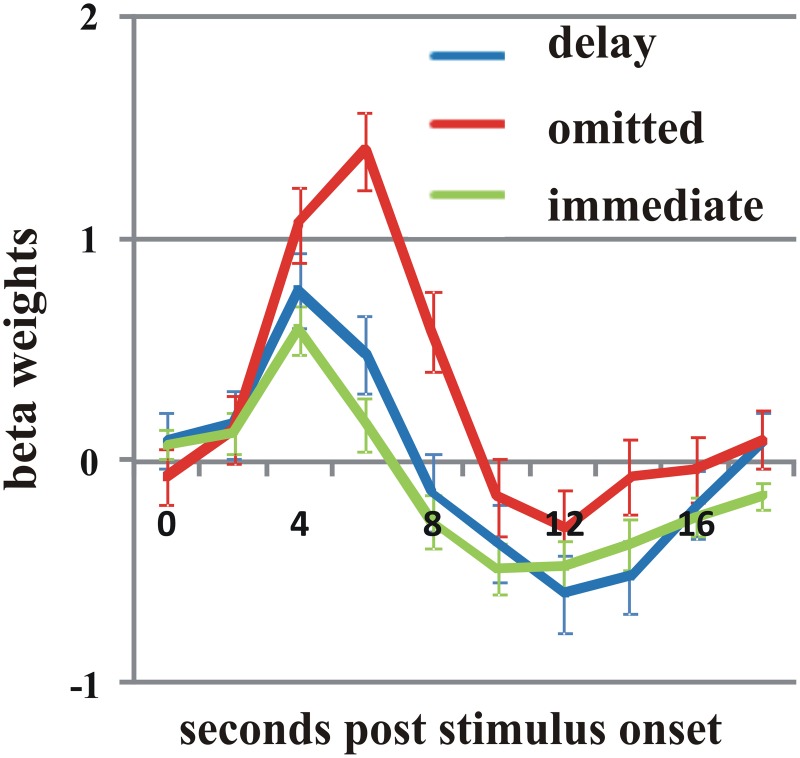
**In both participants who did not notice the delay, omitted feedback (red line) elicited a significantly stronger BOLD response in the posterior medial frontal cortex than delayed feedback (blue line) as well as immediate feedback (green line) (*t* = 3; *p* < 0.003).** Error bars indicate SEM.

As two of the participants reported not to have noticed any delayed feedback, we analyzed them separately and compared their pattern of activity to that of the whole group. Overall, we found a very similar pattern of effects for the delayed and omitted feedback. However, the direct contrast between delayed and omitted feedback revealed in both participants a significant difference in the pMFC with higher activation by omitted than by delayed feedback (Figure 4) (*t* = 3; *p* < 0.003). Furthermore, we found an additional cluster of activation in the bilateral Gfi/aI, with stronger activity by omitted compared to delayed feedback, but only for one of the two subjects.

## Discussion

While we expected significant differences between omitted and delayed feedback due to the fact that the omission of feedback has more serious consequences than a temporally slightly delayed feedback, we only found a few regions more strongly activated during delayed feedback. Besides activation differences in visual areas (inferior occipital gyrus), very likely due to the missing visual stimulus during the omission of feedback, we found a stronger activation during delayed feedback compared to the omission of feedback in the putamen and (in a ROI analysis) in the SN/VTA (Figures 1 and 2). The time course of the BOLD response in these regions revealed a hierarchy of activation strength: the response to the delayed feedback was larger compared to immediate feedback and immediate feedback showed a larger activation compared to the omission of feedback (Tables 2 and 4). This result resembles findings in monkeys, where delayed rewarding feedback resulted in a stronger albeit delayed response of dopaminergic neurons (Hollerman and Schultz, 1998). The result is also consistent with findings in humans that replicate the monkey data in the putamen (McClure et al., 2003) and VTA (D'Ardenne et al., 2008). Even though the omission of registering feedback led to a significant reduction in the BOLD response the deflection was still positive. This is a discrepancy compared to the findings of D'Ardenne et al. (2008) and McClure et al. (2003), who found a negative beta value in response to omitted rewards. The discrepancy may, however, be explained if one assumes that the omission of rewarding feedback reduces the activity to a larger degree than the omission of merely registering feedback, although the mechanism of the reduction may be similar. We want to point out that the reward as used in these studies (McClure et al., 2003; D'Ardenne et al., 2008) not only provides the reward itself but always contains the information that an action by the subject has been registered. Therefore, we argue that already this aspect of feedback leads to a significant activation in dopaminergic structures.

However, one may argue that this simple registering feedback was interpreted as rewarding because it informed the subjects that their motor response successfully elicited a response, i.e., the registering feedback, even though this feedback had no rewarding value in the strict sense. Nevertheless, the dopaminergic system is highly adaptive and it critically depends on the context if feedback is perceived as positive or negative (e.g., Tremblay and Schultz, 1999; Cromwell et al., 2005; Tobler et al., 2005; Kobayashi et al., 2010). Therefore, we assume that feedback which registers a subject's response (law of effect, Thorndike, 1911) may already be sufficient to recruit the dopaminergic system, as already argued in the previous work by Behne et al. (2008). This interpretation is consistent with Zink et al. (2006), who proposed that one function of the striatal response is to reallocate processing resources not only to rewarding but also to non-rewarding unexpected stimuli. Redgrave and Gurney (2006) also assume that a phasic dopamine release after an unpredicted event helps to reselect future actions. We do, however, want to point out that any conclusion as to a potential involvement of dopamine in these processes must be made with great caution because it is currently debated to what extend fMRI allows such conclusions at all (for a recent review see Düzel et al., 2009). For example, the finding in the dorsal striatum may also be explained by its known involvement in basic control of motor responses (e.g., Nakano et al., 2000). Such processes may have been differentially triggered by delayed, omitted, and immediate feedback that provides the user with the information whether an interaction, i.e., a trial has been accomplished.

The direct contrast between the combined unexpected feedbacks and the immediate feedback revealed an equally strong effect in a network of regions, i.e., pMFC, aI/Gfi, dlPFC, Lpi, ACC, and PCC. This result is contrary to our hypothesis that these brain areas should be less strongly activated by delayed feedback compared to the omission of feedback. However, it may reflect general mechanisms of increased attentional demand and adjustments in cognitive and action control, i.e., reorienting responses (Raichle et al., 2001; Kerns et al., 2004; Dosenbach et al., 2006; McKiernan et al., 2006; Taylor et al., 2007; Corbetta et al., 2008) that are already recruited when an expected feedback is delayed by 500 ms. Our results are consistent with a recent finding by Forster and Brown (2011) who also found activation in the pMFC and ACC during unexpectedly late presentation of feedback.

The preliminary finding in two subjects who did not report the occurrence of delayed feedback after the experiment may support this view. While nearly the same network was recruited as in the group, the pMFC showed a differential effect for delayed and omitted feedback with a stronger BOLD response to omitted feedback. This finding is consistent with the idea that the pMFC is activated when a response conflict or unfavorable outcome is indeed detected (Holroyd et al., 2004; Ridderinkhof et al., 2004). Although this interpretation is highly speculative, it seems plausible that events that are not consciously perceived as conflicting or unfavorable do not elicit activity in the pMFC. It remains to be evaluated by testing more subjects why the pMFC is the only brain region in the identified network that was differentially activated when the delay was not noticed.

Due to the oddball design of our feedback presentation one could suggest that the unexpected feedback events might lead to a mismatch negativity (MMN) response. However, the number of areas activated by omitted or delayed compared to immediate feedback as well as the strength of activation speaks against such an argument. The majority of MMN studies using fMRI found corresponding BOLD activity in sensory cortex (Tales et al., 1999; Sabri et al., 2004, 2006; Molholm et al., 2005; Opitz et al., 2005; Kimura et al., 2009). In our study, the visual cortex was not activated by the omitted feedback, only during the delayed feedback. However, we cannot exclude that frontal generators of the MMN response contributed to the activation we found, for example in the pMFC. It is currently under debate whether the ERP component elicited by deviant stimuli and the one elicited by deviant feedback might reflect the same process (Holroyd et al., 2008). Thus, it may be that both reflect a reorientation of attention in response to an unexpected event. However, it remains to be explained why the complete omission of a feedback does not represent a stronger violation of the expectation than a mere delay of the expected feedback by only 500 ms.

In summary, immediate registering feedback elicits activity in dopaminergic midbrain structures. Thus, it may be that the mere registration of a subject's action (e.g., a button press) by “someone” is already valuable information for future actions of a user. In the context of human-computer interaction this is important because it establishes a common ground between human and computer which is a major principle of communication.

Furthermore, in a network of brain regions involved in attention- and action-control, delayed feedback has essentially the same effect as the omission of feedback. Maybe unexpected delays of less than one second already trigger the same neuronal processes that are initiated to adapt one's own behavior to unexpected omissions of feedback. This finding emphasizes the importance of improving the timing of human-computer interactions to prevent the user from wasting cognitive resources while waiting for a feedback.

### Conflict of interest statement

The authors declare that the research was conducted in the absence of any commercial or financial relationships that could be construed as a potential conflict of interest.
